# An icaritin-loaded microemulsion based on coix oil for improved pharmacokinetics and enhanced antitumor efficacy

**DOI:** 10.1080/10717544.2022.2147601

**Published:** 2022-11-29

**Authors:** Huating Zeng, Xiaoqi Li, Yuping Liu, Xia Li, Ding Qu, Yan Chen

**Affiliations:** a Affiliated Hospital of Integrated Traditional Chinese and Western Medicine, Nanjing University of Chinese Medicine, Nanjing, China; b Jiangsu Province Academy of Traditional Chinese Medicine, Nanjing, China

**Keywords:** Icaritin, coix seed oil-based microemulsion, hepatocellular carcinoma, synergistic effect, autophagy

## Abstract

Combinational icaritin (IC) and coix seed oil (CSO) holds promising potential in the treatment of hepatocellular carcinoma. However, traditional cocktail therapy is facing difficulties to optimize the synergistic antitumor efficacy due to the asynchronous pharmacokinetics. Therefore, we developed an icaritin-loaded microemulsion based on coix seed oil (IC-MEs) for improved pharmacokinetics and enhanced antitumor efficacy. The preparation technology of IC-MEs was optimized by the Box–Behnken design and the pharmaceutical properties were characterized in detail. IC-MEs show synergistic antiproliferation against HepG2 cells compared with monotherapy. The mechanism is associated with stronger apoptosis induction via enhancing caspases-3 activity. IC-MEs significantly improve the bioavailability of IC due to the encapsulation of coix oil-based microemulsion and also obtain the desired liver accumulation and elimination. More importantly, IC-MEs exhibit the overwhelming antitumor ability among all of the treatments on the HepG2 xenograft-bearing mice. This study verifies the feasibility of using coix oil-based microemulsion to improve the antitumor effect of water-insoluble components.

## 1. Introduction

Hepatocellular carcinoma (HCC) is the most common and deadliest hepatic malignancy, accounting for the majority of primary liver cancer (Forner et al., 2018; T. Li et al., 2021). For advanced HCC, systemic therapy with sorafenib can be recommended clinically (Kim et al., 2017), but the effect is limited for the patients who are ineffective for chemotherapy and targeted therapy. Therefore, it is imperative to develop a new effective drug for the treatment.

Icaritin (IC) is an active ingredient of prenylflavonoids obtained from *Epimedium* genus (Tan et al., 2016). Clinical data have shown that IC is safe and effective for patients with advanced HCC who are not suitable for chemotherapy and targeted drugs (Fan et al., 2019). However, the low solubility and insufficient drug concentration in liver tissue of IC may limit its antitumor effect, and there is still much room for improvement in its curative effect. Coix seed oil (CSO), extracted from the seed of *Coix lacryma-jobi*, is used for adjuvant treatment of primary liver cancer and nonsmall cell lung carcinoma (X. Huang et al., 2020; Liu et al., 2019). Kanglaite® (CSO) injection is widely used in the clinical adjuvant therapy of cancer. This injection preparation could improve the antitumor effect of the CSO to a certain extent. Nevertheless, for IC, the poor solubility and oral bioavailability limited the full play of its antitumor effect (Zhan et al., 2012; Wu et al., 2018; J. Wang et al., 2021).

Our study found that the combination of IC and CSO performed a synergistic anti-proliferation effect on hepatoma cells. How to achieve the co-loading of two hydrophobic drugs to get a significantly enhanced antitumor effect is a big challenge. Microemulsion, typically prepared from surfactants, co-surfactants, oils, and drugs, has become an effective way to improve the solubility and bioavailability of hydrophobic drugs. In the previous studies, our research group has successfully prepared many microemulsion preparations with co-loading of insoluble components. Therefore, in this article, microemulsion was used as the carrier for the co-loading of IC and CSO, and CSO was used as the oil phase to reduce the amount of excipients. IC and CSO co-loaded microemulsion (IC-MEs) were prepared and the preparation technology was optimized by Box–Behnken design for industrialization. The physicochemical properties including particle size, polydispersity index (PDI), *in vitro* drug release and the stability were characterized. Next, the synergistic antitumor effect of IC-MEs *in vitro* and the mechanism of cell apoptosis were preliminarily explored. Furthermore, the antitumor efficacy *in vivo*, pharmacokinetic characteristics, and tissue distribution of IC-MEs were studied, indicating that IC-MEs could obviously improve the bioavailability and the distribution of IC in tumor tissues.

## 2. Materials and methods

### 2.1. Materials

Icaritin was obtained from Yuanye Biotechnology (purity > 98%, Shanghai, China). Polyoxyl 15 hydroxystearate (HS15) and polyoxyl 40 hydrogenated castor oil (RH40) were purchased from BASF (Ludwigshafen, Germany). Macrogol 400 (PEG400), glyceryl tributyrate were obtained from Sinopharm Chemical Reagent Co., Ltd. (Shanghai, China). CSO was prepared by supercritical CO_2_ extraction by our laboratory and the purity determined by ultraviolet spectroscopy (purity > 85%). 3-Methyladenine (3-MA) was provided by CSNpharm (Chicago, USA). Double-distilled water was acquired by Milli Q purification system. All other reagents were of analytical grade available.

### 2.2. Cell line and animals

Human hepatocellular carcinoma cell (HepG2) was purchased from the Institute of Biochemistry and Cell Biology, Shanghai Academy of Biological Sciences, Chinese Academy of Sciences (Shanghai China).

Male nude mice (BALB/c, 22 ± 2 g) were provided by the Changzhou Cavans Experimental Animal Co., Ltd. (Jiangsu, China). Sprague-Dawley rats (male, 200 ± 20 g) were bought from SPF Biotechnology Co., Ltd. (Beijing, China). All experimental protocols and animal handling procedures were approved by the Animal Ethic Committee of Affiliated Hospital of Integrated Traditional Chinese and Western Medicine, Nanjing University of Chinese Medicine (Approval No.: AEWC20181207-68) and were performed in accordance with institutional, national, and international guidelines for animal care and welfare. All the animals were raised and bred under specific pathogen-free conditions in the Animal Centre of Affiliated Hospital of Integrated Traditional Chinese and Western Medicine, Nanjing University of Chinese Medicine. All mice were kept on a standard 12-h light-to-dark cycle with ad libitum access to food and water, and were anesthetized with 1.5% isoflurane using a Rodent Anesthesia Machine.

### 2.3. Preparation of IC-MEs

Based on our previous study, CSO–glyceryl tributyrate (a ratio of 5:1, wt:wt), HS15–RH40 (a ratio of 2:1, wt:wt), and PEG400 were respectively selected as the mixed oil phase, mixed surfactant, and co-surfactant of microemulsion (Zeng et al., 2020). IC-MEs were prepared as follows: First, IC and excipients were magnetically stirred at room temperature. Then, double-distilled water was slowly added into the mixture with magnetic stirring to obtain a clear and transparent IC-MEs solution (Chen et al. 2018). To achieve the industrialization of IC-MEs, Box–Behnken design was applied to optimize the preparation parameters of IC-MEs (Mohammad et al., 2019; Cheng et al., 2020). Three components were included as independent variables: stirring speed (*X*_1_), stirring time (*X*_2_), dropping water speed (*X*_3_), which were set within ranges of 150–350 rpm, 2–6 h, 25–60 mL/min. Two response variables including particle size (*Y*_1_) and PDI (*Y*_2_) were adopted.

### 2.4. Characterization of IC-MEs

The particle size, PDI, and zeta potential of IC-MEs were measured by a laser particle analyzer (Malvern, Nano-ZS 3600, UK).

The diluted IC-MEs were taken on a copper grid and stained with uranyl acetate followed by drying at room temperature. The morphology of IC-MEs was visualized with transmission electron microscopy (TEM, HT7700, Hitachi, Tokyo, Japan).

The encapsulation efficiency (EE) was calculated according to the following equation:

$$\text{EE}\;\left(\%\right)\;=\;W_{\text{encapsulated}\;\text{drug}}/W_{\text{feeding}\;\text{drug}}\times \;100\%$$

where *W*_feeding drug_ and *W*_encapsulated drug_ represent the amount of IC in the microemulsion before and after ultrafiltration centrifugation separation, respectively.

The content of IC was evaluated by high-performance liquid chromatography (Waters Acquity ARC, Massachusetts, USA). The chromatographic conditions were as follows: the column was Elite ODS2 column (4.6 mm × 150 mm, 5 μm) (Elite, Dalian, China), the mobile phase was acetonitrile and 0.1% acetic acid aqueous solution (85:15), the flow rate was 1.0 mL/min, the detection wavelength was 270 nm, the column temperature was 30 °C, and the injection size was 10 μL.

### 2.5. *In vitro* drug release assay

*In vitro* drug release assay of IC-MEs was performed by dialysis bag method as described in our previous report (Qu et al., 2018). Briefly, 1 mL of IC-MEs solution was placed in a dialysis bag (MWCO: 10 kDa) and immersed in 120 mL of phosphate buffered saline at pH 7.4 and 6.4 containing 0.5% vol/vol Tween 80 at 37 ± 0.5 °C with constantly shaking at a speed of 50 rpm. Samples of 20 µL were collected at 0.25, 0.5, 1, 2, 4, 6, 8, 12, and 24 h respectively and 20 µL of corresponding fresh medium was replaced instantly. The sample was diluted with 300 μL methanol followed by centrifugation with 13,000 rpm. Next, IC in the supernatant was analyzed by HPLC and the chromatographic conditions are the same as those in Section 2.4.

The drug release kinetics was investigated by zero-order, first-order, Higuchi diffusion model, and Ritger–Peppas equation.

### 2.6. Stability assay

The stability of IC-MEs was investigated under the conditions of high-speed centrifugation and cool–heat cycle. After centrifugation at 10,000 rpm for 5 min, drug precipitation, droplet combination, emulsification, and phase separation of microemulsion were observed, and the particle size and PDI were determined. The temperature stability of IC-MEs was tested by three cold and hot cycles between 4 (±2 °C) and 25 (±2 °C), and the storage stability of IC-MES in 4 weeks was evaluated by checking particle size and PDI.

### 2.7. *In vitro* antitumor efficacy

#### 2.7.1. Cellular uptake assay

In the study of cell uptake, coumarin 6 (C6) was employed as a fluorescence probe to visualize the intracellular microemulsion. To evaluate the internalization, Coumarin6 (C6) was used as a probe to label IC-MEs (C6/IC-MEs), and free C6 was used as controls. HepG2 cells were incubated into a 6-well plate at a density of 2 × 10^5^ cells/well for 24 h, and then incubated with C6/IC-MEs and free C6 for 2 h, respectively. The concentration of C6/IC-MEs and free C6 was calculated as 100 ng/mL of C6. The cells were washed by ice-cold PBS and immediately observed under fluorescence microscope (Olympus, Tokyo, Japan). The cells rinsed by ice-cold PBS were trypsinized by trypsin without EDTA to obtain cell suspension. Finally, the cells were suspended in 0.2 mL of PBS and immediately analyzed by flow cytometry (Beckman Coulter, Inc., Miami, USA).

To investigate the cellular mechanisms of IC-MEs, HepG2 cells with endocytic inhibitors or at 4 °C were applied. The cells were respectively treated with 1 mL of genistein (54 μg/mL), sucrose (154 μg/mL), and amiloride (133 μg/mL) at 37 °C for 1 h, then treated with 1 mL of DMEM at 4 °C for 1 h, and then incubated with C6/IC-MEs for 2 h. Next, fluorescence intensity of the cells was detected by flow cytometry. All of the experiments were performed in triplicates.

#### 2.7.2. Anti-proliferation and synergistic effect *in vitro* assay

3-(4,5-dimethyl-2-thiazolyl)-2,5-diphenyl-2-H-tetrazolium bromide,Thiazolyl Blue Tetrazolium Bromide (MTT) assay was used to investigate the anti-proliferation effect of IC-MEs on HepG2 cells and synergistic antitumor effect of CSO and IC. HepG2 cells (5 × 10^4^ per well) were cultured in a 96-well plate for 24 h. IC, CSO, mixture of IC + CSO, and IC-MEs (35.00, 17.50, 8.75, 4.38, 2.19, 1.09 μg/mL per group with IC concentration) were administered 100 μL per well to the treatment group. The control group was given 100 μL DMEM incomplete medium. After the culture was continued for 24 h, 10 μL MTT (5 mg/mL) was added into each well in the dark condition, followed by incubation for 4 h. Then 100 μL DMSO solution was added into each well, and the absorbance (*A*) of each well was measured at 490 nm by Microplate Reader (Thermo Fisher Scientific, Massachusetts, USA). Cell viability (%) was calculated by comparing the absorbance of various groups (OD_sample_) to that of the wells with DMEM (OD_control_) using the following formula:

$$\text{Cell}\;\text{viability}\;\left(\%\right)\;=\;\left[\left({\text{OD}}_{\text{sample}}\right)/\left({\text{OD}}_{\text{control}}\right)\right]\;\times \;100\%$$

The cell half inhibitory concentration (IC_50_) was calculated by Graphpad Prism 7.0 software. combination index (CI) applied to evaluate the synergistic antitumor effect of CSO and IC was calculated by following equation:

$$\text{CI}\;={\;\text{IC}}_{50}^{a}{/\text{IC}}_{50}^{1}+{\;\text{IC}}_{50}^{b}/{\text{IC}}_{50}^{2}$$

where ${\text{IC}}_{50}^{1}$ and ${\text{IC}}_{50}^{2}$ are the IC_50_ of IC and CSO respectively, ${\text{IC}}_{50}^{a}$ and ${\text{IC}}_{50}^{b}$ are represented by the IC_50_ of IC and coix seed oil of IC-MEs or mixture, respectively. The synergistic effect is considered as CI < 1.

#### 2.7.3. Cell apoptosis induction

The cell apoptosis of IC, CSO, mixture of IC + CSO, and IC-MEs against HepG2 cells was evaluated using Annexin V-FITC/propidium iodide (PI) detection Kit (KeyGEN Bio, Nanjing, China). HepG2 cells were plated at a 6-well plate at a density of 1 × 10^5^/well into an incubator for 24 h, and then incubated with IC, CSO, mixture of IC + CSO, and IC-MEs for 24 h, respectively. The supernatant and cells were collected according to the protocol. The cells were analyzed immediately using the flow cytometry.

#### 2.7.4. Induction of autophagy in HepG2 cell

To explore the antitumor mechanism of IC-MEs in HepG2 cells, different concentrations of IC-MEs (16 μg/mL, 8 μg/mL, and 4 μg/mL) and various formulations (including IC, CSO, mixture of IC and CSO, and IC-MEs) were investigated. HepG2 cells were seeded at a density of 2 × 10^6^ per well in 10 cm cell culture dish and incubated overnight. HepG2 cells were incubated with IC, CSO, mixture of IC + CSO, and IC-MEs for 24 h, then the protein was extracted and the expression of LC3 protein in each administration group was detected by western blot. SDS-PAGE Gel Quick Preparation Kit was used for the western blot assay (Abcam, Cambridge, UK).

### 2.8. Antitumor efficacy evaluation *in vivo*

The mixed suspension of HepG2 (1 × 10^7^) and LX-2 cells (1 × 10^7^) with a total volume of 0.2 mL was injected subcutaneously into the right axilla of each nude mouse. When the subcutaneous ectopic tumors of the mice grew to 100–200 mm^3^, the nude mice were randomly divided into normal saline group, IC group, CSO group, and IC-MEs group. Every other day, 2.5 mg/kg of various IC groups or 100 mg/kg of CSO groups were injected intraperitoneally. During the experiment, the state of nude mice was observed daily and the body weight of nude mice in each group was weighed. The long diameter (*A*) and the short diameter (*B*) of the tumor were measured with a vernier caliper, and the volume of the tumor was calculated according to the formula:

$$V=\left(a\;\times \;b^{2}\right)/2.$$

At 24 days of post-xenograft implantation, the tumors were collected after mice were euthanized. The tumor was weighted, and the tumor inhibition of each administration group was calculated according to the following formula:

Tumor index of the model group=tumor weight (mg) of the normal saline group/body weight of the nude mouse (g)

Tumor inhibition rate (%)=(tumor index of the model group−tumor index of the administration group)/tumor index of the model group×100%

### 2.9. Pharmacokinetic study

#### 2.9.1. Sample collection

Twelve male Sprague-Dawley (SD) rats were randomly divided into two groups that were IC and IC-MEs groups (*n* = 6). Before the treatment, SD rats were fasted for 12 h and were exposed to water freely. IC and IC-MEs were injected intraperitoneally at a dose of 60 mg/kg, respectively. At the interval of 0.033, 0.083, 0.25, 0.5, 0.75, 1, 2, 4, 6, 8, 12, and 24 h after administration, 250 µL of blood samples were collected from the retinal venous plexus of rat and placed into a centrifuge tube containing heparin sodium. Plasma samples were collected by centrifugation at 3500 rpm for 15 min at 4 °C and stored at –80 °C for subsequent analysis.

IC was extracted from rat plasma by a validated method. Briefly, 150 µL of plasma sample was extracted with 750 µL of ethyl acetate. The collected supernatant was evaporated to dryness under nitrogen gas and then reconstituted in 200 µL of methanol. The supernatant was quantitatively analyzed by HPLC method coupling online to Quadrupole Dalton analyzer (QDa).

#### 2.9.2. HPLC–QDa analysis

An analytical HPLC–QDa method was developed to determine the concentration of IC. The chromatographic conditions were: the column was Elite ODS2 column (4.6 mm × 150 mm, 5 μm) (Elite, Dalian, China), the mobile phase was acetonitrile and 0.1% acetic acid aqueous solution (70:30), the flow rate was 0.8 mL/min, the detection wavelength was 270 nm, and the column temperature was 30 °C. The QDa condition was as follows: the capillary pressure was 0.8 kV, the cone hole voltage was 30 V, the sampling speed was 5 point/s, and the mass/charge ratios (*m*/*z*) of icaritin and genistein (internal standard substances, IS) were 367 and 269, respectively.

#### 2.9.3. Analytical method validation

Under the above chromatographic condition, the interference of endogenous substances was not observed in plasma and all tissue samples, indicating that this method had good specificity. In addition, the intra-day and inter-day precision, accuracy, extraction recovery, and sample stability were evaluated by three different concentrations (low, medium, and high) of quality control (QC) samples in rat plasma.

#### 2.9.4. Pharmacokinetic parameters and *in vivo* and *in vitro* correlation analysis

The main pharmacokinetic parameters were calculated by non-compartmental model analysis based on Drug and Statistics 2.0 (DAS 2.0, Chinese Pharmacological Society).

*In vivo* and *in vitro* correlation (IVIVC) is the relationship between *in vitro* release behavior and *in vivo* pharmacokinetics, and could be described by a mathematical model. In this study, IVIVC of IC-MEs was studied by the curves of cumulative release rate *in vitro* and absorption percentage *in vivo* at different time points within 24 h. The IVIVC equation was established with the cumulative release rate *in vitro* as the independent and the absorption fraction *in vivo* as the dependent variable. The cumulative release rate could be obtained from the above drug release assay *in vitro*, and the absorption fraction (Fa) *in vivo* was expressed as follows:

$${\text{Fa}}_{（t）}={\;\text{AUC}}_{0-t}/{\text{AUC}}_{0-\infty }\times \;100\%$$

where Fa_(_*_t_*_)_ is the percentage of drug absorbed over time *t*, AUC_0-_*_t_* is the area of blood concentration over time *t*.

### 2.10. Tissue distribution study

#### 2.10.1. Sample collection

Seventy-two SD rats were randomly divided into two groups that were IC and IC-MEs groups (*n* = 36). Before the experiment, SD rats were fasted for 12 h and were exposed to water freely. IC and IC-MEs were injected intraperitoneally at a dose of 60 mg/kg, respectively. At 1, 2, 4, 6, 12, 24 h after administration, rats were humanely sacrificed and then tissues (heart, liver, spleen, lung, kidney, and brain) were collected, washed with ice-cold saline and dried with filter paper. The tissue samples were stored at –80 °C until analysis.

#### 2.10.2. Sample analysis

An effective method to extract IC from rat tissue was developed. Briefly, 0.5 mL tissue homogenate (0.5 g/mL, wt/vol) was extracted with 2.5 mL of ethyl acetate. The collected supernatant was evaporated to dryness under nitrogen, and then reconstituted in 200 µL of methanol, followed by centrifugation. Next, the sample was determined by HPLC-QDa. The chromatographic conditions and analytical methods are the same as those in Sections 2.9.2 and 2.9.3.

### 2.11. Safety evaluation

After 24-day post-xenograft implantation, the major organs including heart, liver, spleen, lung, and kidney were collected after euthanasia and fixed by formalin. Then these fixed organs tissues were embedded in paraffin blocks for H&E and visualized by optical microscope (Olympus, Japan) for pathological safety evaluation.

### 2.12. Statistical analysis

All data were expressed as mean ± standard deviation and the difference between the groups was analyzed using the *t* test. For all statistical comparisons, the values of *p < .05, **p < .01, ***p < .001 were considered statistically significant.

## 3. Results and discussion

### 3.1. Preparation and characterization of IC-MEs

IC, which belongs to the group of BCS (biopharmaceutics classification system) II components, has good intestinal permeability, but its poor water solubility (<0.2 μg/mL) and bioavailability (35% in rats) have hindered its future application. An appropriate drug delivery system is necessary for IC to develop its clinical use. Microemulsion is a promising nanoscale drug delivery system, which can effectively improve the water solubility and bioavailability of drugs. Meanwhile, its preparation process is simple and easy for industrialization.

**Figure 1.**
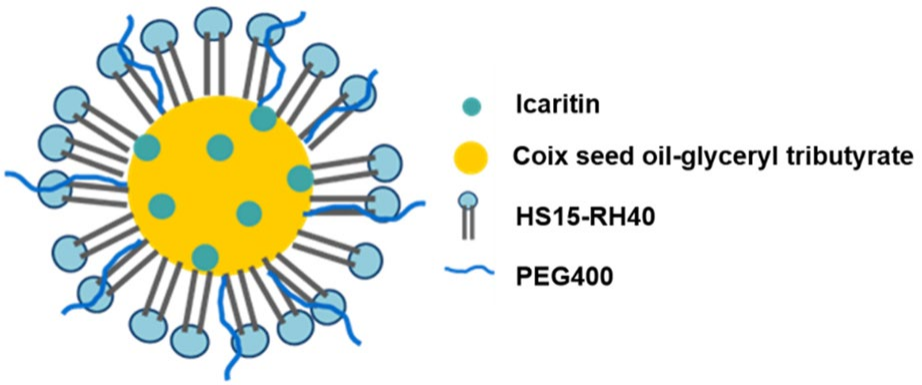
Structural design of IC-MEs.

In previous research, we found that CSO can be used as the oil phase of microemulsion, which can reduce the dosage of excipients, and have a synergistic anticancer effect (Qu et al., 2015). A series of CSO microemulsion systems were constructed and used for the delivery of undissolvable/insoluble drugs (such as tripterine). Therefore, in this study, CSO was still used as the oil phase in microemulsion co-loading IC (Figure 1). In our early study, the formulation of MEs was optimized. CSO–glyceryl tributyrate (a ratio of 5:1, wt:wt) were used as the mixed oil phase of IC-MEs, while HS15–RH40 (a ratio of 2:1, wt:wt) and PEG400 were respectively applied as the mixed surfactant and co-surfactant of IC-MEs in the small trial study on prescription of preparation (Zeng et al., 2020). The best ratio of IC and mixed oil phase was determined as 1:30 (wt/wt).

**Figure 2.**
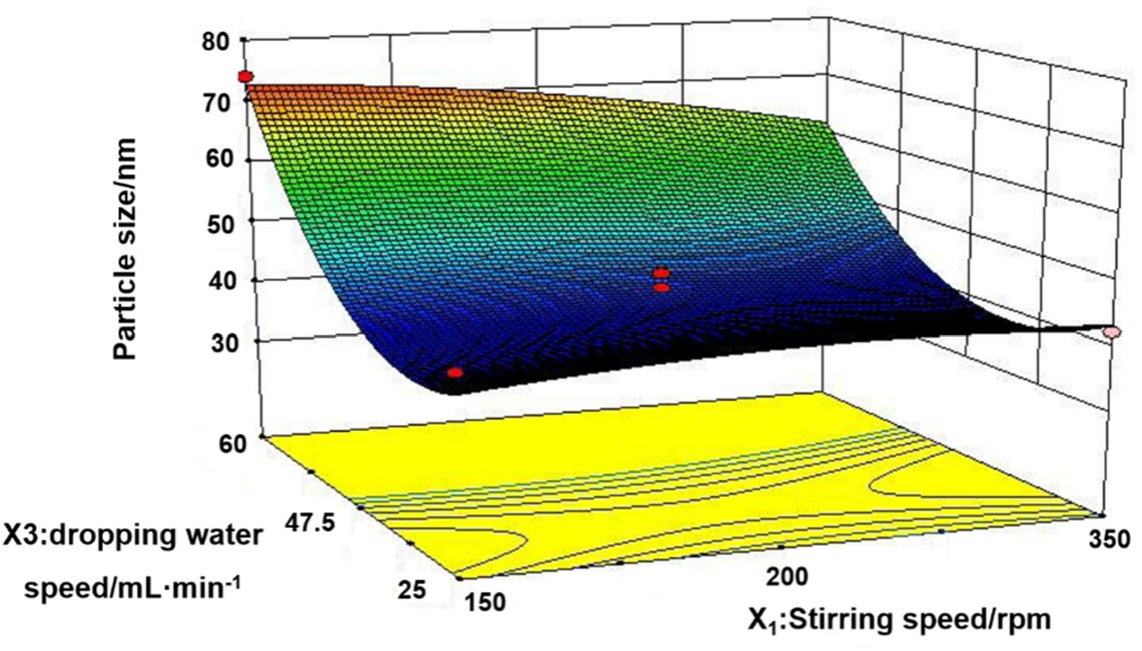
3-D response surface plot of effective parameters on particle size.

**Figure 3.**
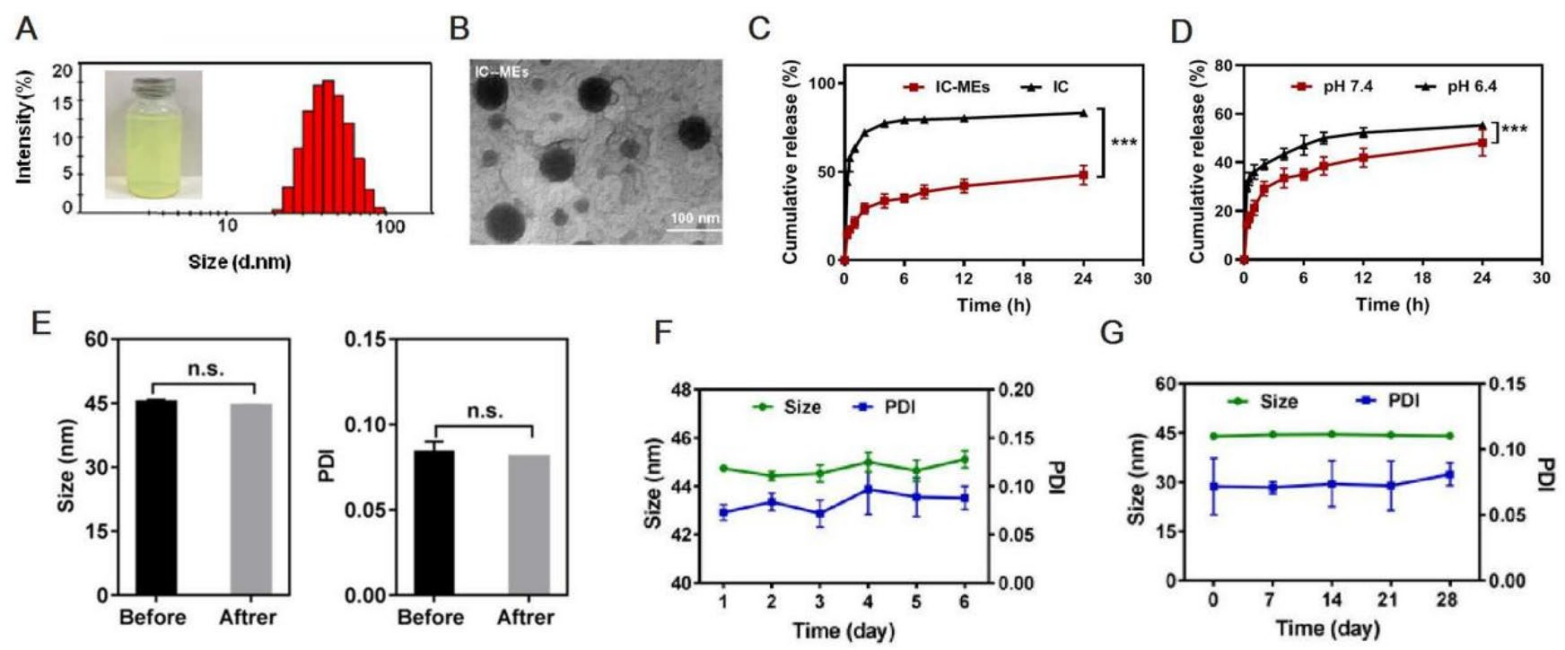
Characterization of IC-MEs. (A) Appearance and particle distribution of IC-MEs. (B) TEM image of IC-MEs. (C) *In vitro* release profile of IC-MEs and IC under physiological pH condition. *n* = 3, ***p < .001, IC-MEs vs. IC. (D) *In vitro* release profile of IC-MEs at various pH environments. Data are represented as mean ± SD, *n* = 3, ***p < .001, pH 6.4 vs. pH 7.4. (E) Stability evaluation of IC-MEs with high-speed centrifugation. (F) Stability evaluation of IC-MEs with heating/ cooling cycles. (G) Stability evaluation of IC-MEs with storage time. Data are represented as mean ± SD, *n* = 3, *p < 0.05.

To promote the industrial production of microemulsion, Box–Behnken design (Table 1) was used to screen the three main process parameters of microemulsion, namely, stirring speed (*X*_1_), stirring time (*X*_2_), and dropping water speed (*X*_3_). Only the dropping water speed had a significant effect (*p* < .001) on the particle size and PDI of microemulsion (Tables 2 and 3). Analysis of variance for the fitted model also revealed that the binary interaction of stirring speed and dropping water speed have great influence (*p* < .05) on the size of microemulsion. At a constant level of stirring time, the observed increase in the size of particles followed by the increase of dropping water speed and the decrease of stirring speed was represented in 3-D response surface plot (Figure 2). After comprehensive evaluation by Design Expert 8.0.5 software, the optimal process conditions were as follows: stirring speed of 350 rpm, stirring time of 2 h, and dropping water speed of 42.5 mL/min. Under the optimal parameters, a pilot scale-up experiment was conducted. As shown in Figure 3(A), the prepared microemulsion was a clear, transparent liquid with a faint yellow opalescence, and the average particle size was 41.82 ± 0.42 nm, with a PDI of 0.078 ± 0.009, which was consistent with the results of the small trial test. The small particle size suggested that the enhancement of the enhanced permeability and retention (EPR) effect of IC-MEs might deeply penetrate to the tumor tissue and better exert the curative effect. The small PDI value indicated that IC-MEs had uniform particle size and was suitable for industrialization. According to TEM characterizations (Figure 3(B)), IC-MEs displayed a spherical homogeneous morphology with the similar particle size determined by dynamic light scattering (DLS). Taken together, the preparation process of microemulsion was simple and suitable for industrial production.

**Table 1.** Box–Behnken design.

	Factors			Particle size (*Y*_1_, nm)		PDI (*Y*_2_)	
No.	*X*_1_	*X*_2_	*X*_3_	Predictive	Experimental	Predictive	Experimental
1	0	–1	–1	45.44	44.68 ± 0.25	0.101	0.099 ± 0.010
2	0	0	0	43.95	42.23 ± 0.26	0.087	0.070 ± 0.003
3	–1	0	–1	41.82	43.70 ± 0.19	0.077	0.077 ± 0.010
4	–1	1	0	44.08	42.13 ± 0.31	0.087	0.088 ± 0.009
5	0	0	0	43.95	46.32 ± 0.59	0.087	0.101 ± 0.012
6	–1	0	1	72.83	74.05 ± 0.53	0.231	0.228 ± 0.008
7	1	–1	0	41.82	41.82 ± 0.42	0.086	0.078 ± 0.009
8	0	1	1	70.90	71.65 ± 0.68	0.229	0.231 ± 0.007
9	0	0	0	43.95	43.04 ± 0.52	0.087	0.093 ± 0.021
10	1	0	–1	43.38	42.19 ± 0.24	0.080	0.084 ± 0.017
11	0	0	0	43.95	43.71 ± 0.32	0.087	0.090 ± 0.018
12	0	1	–1	43.55	42.38 ± 0.42	0.072	0.066 ± 0.005
13	1	0	1	61.84	58.72 ± 0.65	0.194	0.189 ± 0.009
14	–1	–1	0	45.83	43.47 ± 0.14	0.091	0.088 ± 0.010
15	1	1	0	41.82	44.18 ± 0.36	0.070	0.073 ± 0.012
16	0	–1	1	68.81	69.98 ± 0.77	0.216	0.222 ± 0.003
17	0	0	0	43.95	44.45 ± 0.06	0.087	0.080 ± 0.009

*X*_1_: stirring speed (rpm), *X*_2_: stirring time (h), *X*_3_: dropping water speed (mL/min).

Data are represented as mean ± SD, *n* = 3.

**Table 2.** ANOVA for size according to response surface quadratic model.

	Analysis of variance table				
Parameter	Sum of squares	df	Mean square	*F* value	*p* value
Model	2045.40	9	227.27	29.14	<.0001
*X*_1_	33.78	1	33.78	4.33	.0759
*X*_2_	0.019	1	0.019	2.438 × 10^-3^	.9620
*X*_3_	1286.51	1	1286.51	164.95	<.0001
*X*_1_ *X*_2_	3.42	1	3.42	0.44	.5289
*X*_1_ *X*_3_	47.75	1	47.75	6.12	.0426
*X*_2_ *X*_3_	3.94	1	3.94	0.51	.5002
$X_{1}^{2}$	13.32	1	13.32	1.71	.2325
$X_{2}^{2}$	2.24	1	2.24	0.29	.6089
$X_{3}^{2}$	657.24	1	657.24	84.27	<.0001
Residual	54.60	7	7.80		
Lack of fit	44.88	3	14.96	6.16	.0557
Pure error	9.71	4	2.43		
Cor Total	2099.99	16			

**Table 3.** ANOVA for PDI according to response surface quadratic model.

Parameter	Analysis of variance table				
	Sum of squares	df	Mean square	*F* value	*p* value
Model	0.057	9	6.289 × 10^–3^	58.07	<.0001
*X*_1_	4.061 × 10^–4^	1	4.061 × 10^–4^	3.75	.0940
*X*_2_	1.051 × 10^–4^	1	1.051 × 10^–4^	0.97	.3573
*X*_3_	0.037	1	0.037	341.59	<.0001
*X*_1_ *X*_2_	6.250 × 10^–6^	1	6.250 × 10^–4^	0.058	.8170
*X*_1_ *X*_3_	5.290 × 10^–4^	1	5.290 × 10^–4^	4.88	.0628
*X*_2_ *X*_3_	4.410 × 10^–4^	1	4.410 × 10^–4^	4.07	.0834
$X_{1}^{2}$	2.384 × 10^–4^	1	2.384 × 10^–4^	2.20	.1814
$X_{2}^{2}$	2.579 × 10^–5^	1	2.579 × 10^–4^	0.24	.6404
$X_{3}^{2}$	0.018	1	0.018	165.41	<.0001
Residual	7.581 × 10^–4^	7	1.083 × 10^–4^		
Lack of fit	1.793 × 10^–4^	3	5.975 × 10^–4^	0.41	.7533
Pure error	5.788 × 10^–4^	4	1.447 × 10^–4^		
Cor Total	0.057	16			

### 3.2. *In vitro* release and stability of IC-MEs

To evaluate the *in vitro* release behavior of IC-MEs, we have compared the release profiles of IC-MEs and free IC under physiological pH condition, and investigated the release profiles of IC-MEs under physiological and tumor microenvironment conditions by analytical bag method. In this study, the pH values of 7.4 and 6.4 simulated that of the physiological environment and tumor microenvironment respectively. Drug contents at different time points were determined by HPLC, and the *in vitro* release curve was drawn. As shown in Figure 3(C), the cumulative release of IC-MEs reached 42% at 12 h, while the cumulative release of free IC reached 44% at 0.25 h. The obviously prolongation of the circulation time in blood of IC-MEs may improve bioavailability of IC and lead to the better antitumor effect of IC-MEs. According to Figure 3(D), when pH decreased to 6.4, the release rate of IC-MEs increased by 30% for 12 h. This significant increase in release was probably owing to the different stability of coix seed oil under different pH conditions. The decrease in pH resulted in the hydrolysis of glycerol, the main ingredient of coix seed oil. With the destruction of oil phase structure of microemulsion, the permeability of IC-MEs would also change (Qu et al., 2015). The increased release of the microemulsion at pH6.4 indicated that IC-MEs may have a certain responsive release ability to the tumor microenvironment, which can improve the killing effect of IC on liver tumors and reduce the toxic and side effect on normal tissues to a certain extent.

Next, we have evaluated the release mechanism of IC-MEs by three release models including zero-order equation, first-order equation, and Higuchi equation (Y. Huang et al., 2018). The correlation coefficient (*R*^2^) of the equation represents the fitting degree. According to the results shown in Table 4, the *in vitro* release behavior of IC-MEs had the highest fitting degree with Higuchi equation while its *R*^2^ was 0.9163. In addition, the release mechanism of IC-MEs was further verified by Ritger–Peppas equation (Table 4). The release index *n* of the Ritger–Peppas equation represents a characteristic parameter of drug release mechanism. When *n* is not more than 0.43, the drug release mechanism is Fickian diffusion. When *n* is greater than 0.43 and less than 0.85, the drug release mechanism is non-Fickian diffusion, which represents the combined effect of diffusion and framework dissolution. When *n* is greater than or equal to 0.85, the drug release mechanism is framework dissolution. As illustrated in Table 4, the release index of IC-MEs was 0.27, which mainly indicated that the release mechanism of IC-MEs was Fickian diffusion.

**Table 4.** *In vitro* release kinetics of IC-MEs.

Model	Equation	*R* ^2^
Zeo-order	*Q_t_* = 1.29*t* + 22.74	0.7144
First-order	*Q_t_* = 39.62(1 – *e*^–0.85^*^t^*)	0.7921
Higuchi	*Q_t_* = 7.68*t*^1/2^ + 14.61	0.9163
Ritger–Peppas	*Q_t_* = 21.50*t*^0.27^	0.9869

In order to investigate the stability of IC-MEs, the size and PDI of IC-MEs were recorded using various intervention methods, including high-speed centrifugation, heat–cool cycle, and storage at 4 weeks. As shown in Figure 3(E), both of IC-MEs had stable size range around 44 nm before and after high-speed centrifugation. In addition, we did not observe significant change of size, PDI, or precipitate under heating/cooling cycles (Figure 3(F)), representing that IC-MEs had an acceptable thermostability. After storage within 4 weeks, the size of IC-MEs maintained about 45 nm (Figure 3(G)). Taken together, IC-MEs had a good stability for the industrial production.

### 3.3. Antitumor efficacy *in vitro*

#### 3.3.1. Cellular uptake study

**Figure 4.**
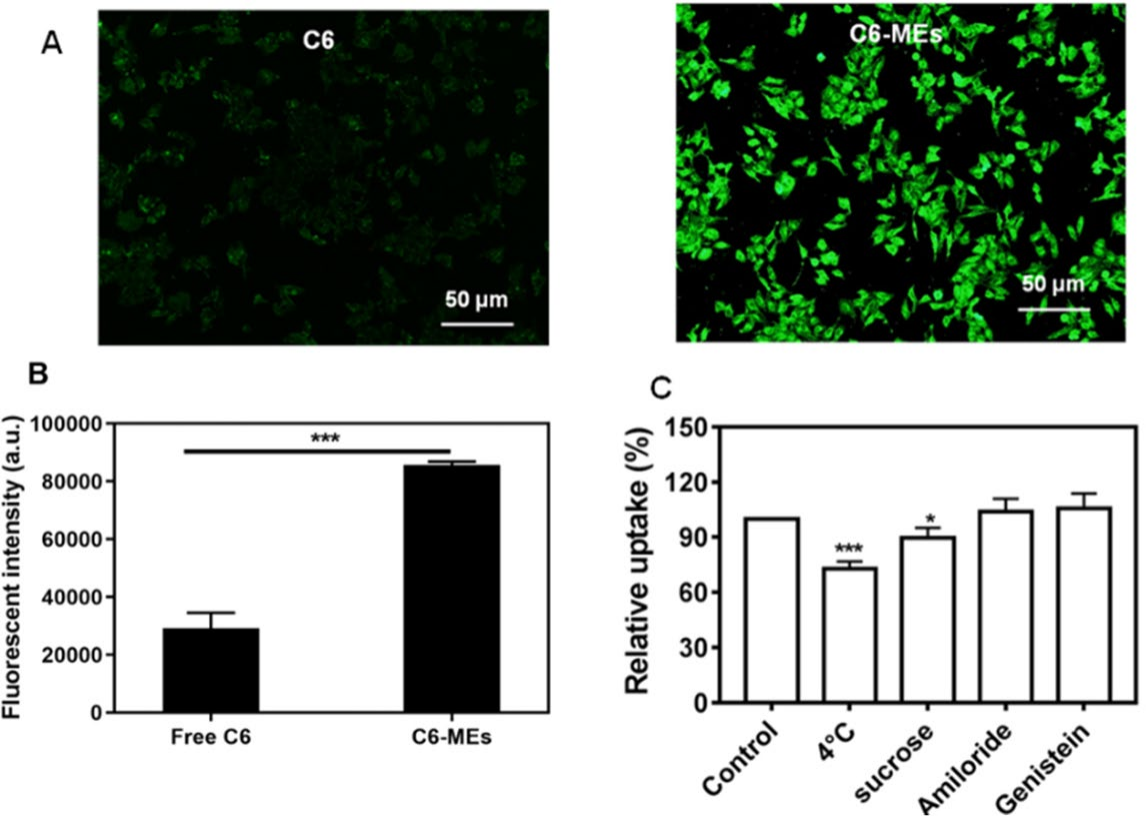
*In vitro* cellular uptake studies. (A) Fluorescent images of HepG2 cells incubated with C6/IC-MEs and C6. (B) Fluorescence intensity of HepG2 cells incubated with C6/IC-MEs and C6 measured by flow cytometry. Data are represented as mean ± SD, *n* = 3, ***p < .001. (C) Uptake mechanism study of C6/IC-MEs. Data are represented as mean ± SD, *n* = 3, compared with control group, *p < .05, ***p < .001.

To evaluate the cellular uptake of IC-MEs, we investigated the intracellular fluorescence of HepG2 cells treated with free C6 and C6 labeled IC-MEs (C6/IC-MEs), respectively. As shown in Figure 4(A), the intracellular green fluorescence of C6/IC-MEs group was significantly higher than that of free C6 group. In the quantitation of intracellular fluorescent intensity, similar results were also obtained by flow cytometry. Notably, the fluorescent intensity of C6/IC-MEs was almost three times as high as that of free C6 (Figure 4(B)), indicating that CSO-based microemulsion was beneficial for drug internalization.

The uptake mechanism of IC-MEs was further studied. As shown in Figure 4(C), the cellular uptake of C6/IC-MEs was significantly inhibited at 4 °C, which indicated that the endocytosis of C6/IC-MEs in HepG2 cells was energy-dependent. Furthermore, the competitive uptake inhibitors of sucrose, genistein, and amiloride were applied to specifically suppress clathrin-associated endocytosis, caveolae-mediated endocytosis, and macropinocytosis, respectively. The cellular uptake of C6/IC-MEs was significantly decreased after the pretreatment with sucrose, indicating that the internalization of the CSO-based microemulsion was mediated by clathrin-associated pathway.

#### 3.3.2. Anti-proliferation and synergistic antitumor effect *in vitro*

CSO, as the excipient and anti-tumor active ingredient in IC-MEs, could be combined with other chemotherapeutic drugs or active ingredients of traditional Chinese medicines (tripterine, norcantharidin, and etoposide) to exert synergistic antitumor effect (Qu et al. 2015; D. Wang et al., 2017; Guo et al., 2019). IC and CSO both have antitumor and auxiliary antitumor effects when used alone in clinic. Whether they have synergistic antitumor effects has not been reported yet. This study is the first time to explore the synergistic antitumor effects of the two drugs and IC-MEs.

**Figure 5.**
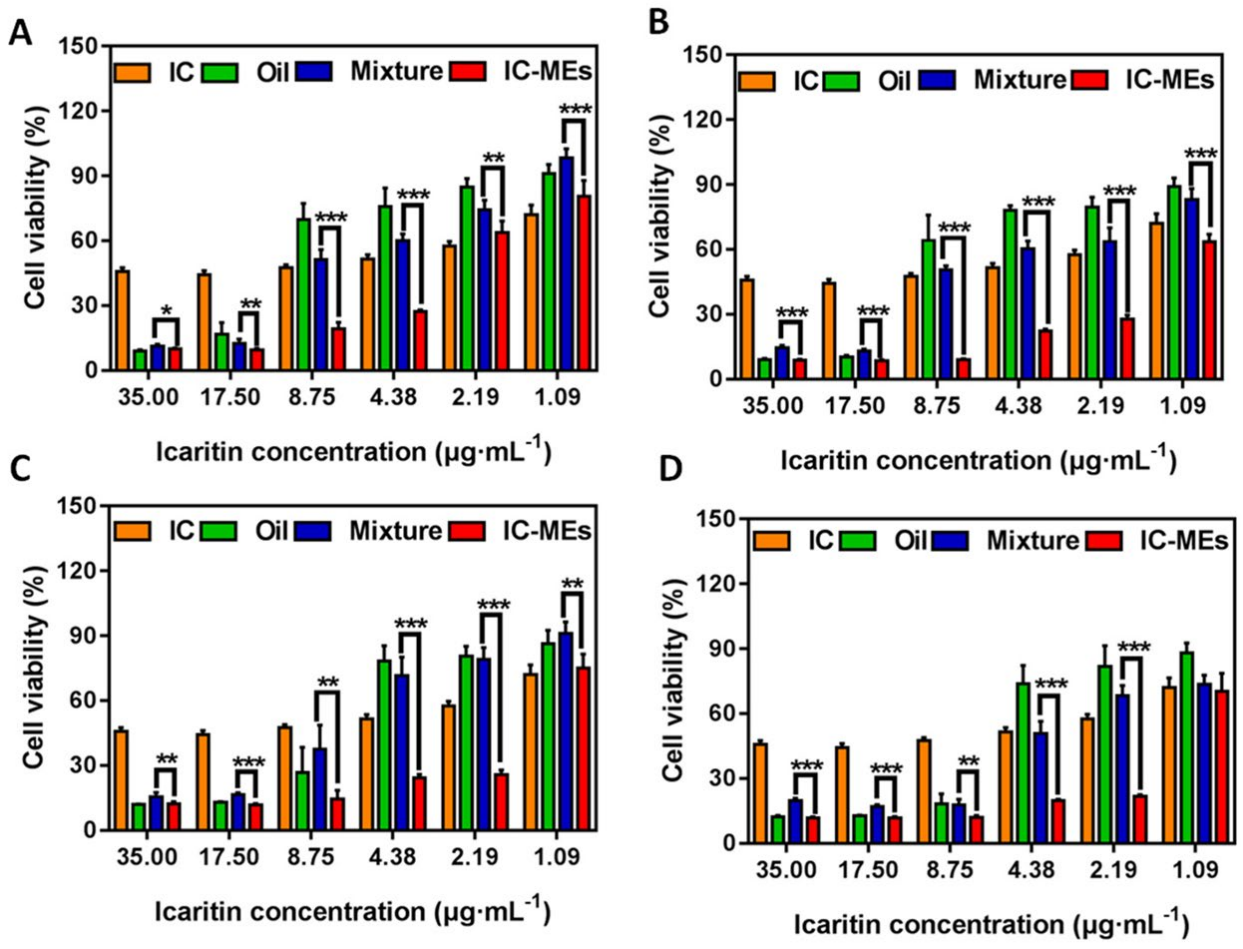
Cytotoxicity of IC-MEs prepared with different ratio of IC and CSO, (A): 1:20, (B): 1:30, (C): 1:40, (D): 1:50 ($\bar{x}$*x* ±* s*, *n* = 6, ***p < .001, **p < .01, *p < .05).

Figure 5 shows the cell viability of HepG2 cells after treatment with CSO and three IC-based formulations. Four weight ratios of IC and CSO (1:20, 1:30, 1:40, 1:50, wt:wt) were studied. The cell viability of each group decreased when the concentration of IC increased from 1.09 μg/mL to 35.00 μg/mL. Under the same weight ratio of IC to CSO, the cell viability of IC-MEs group was significantly lower than that of the mixture of IC + CSO group (*p* < .05), which suggested that IC-MEs had a strong anti-proliferation effect on HepG2 cells. To further evaluate the synergistic antitumor effect of IC-MEs, the half maximum inhibitory concentration (IC50) and the CI were examined (Table 5). A CI value of less than 1 indicated a synergistic antitumor effect. The smaller the CI was, the stronger the synergistic antitumor effect (Guo et al. 2019; Zhong et al., 2017; Han et al., 2012). As shown in Table 5, IC-MEs significantly reduced the IC50 of IC (*p* < 0.05) and the CI values of IC-MEs were all less than 1, showing that IC-MEs had an obvious synergistic antitumor effect. Among the four weight ratios of IC and CSO, 1:30 (wt:wt) manifested the strongest synergistic effect (CI = 0.31) compared with other groups. The above results suggested that the significantly enhanced synergistic antitumor effect of IC-MEs might be relevant to the increased uptake of IC by microemulsion.

**Table 5.** IC_50_ and CI of IC and CSO in various formulations against HepG2 cells.

Formulations	IC_50_ (μg/mL)		CI
	IC	CSO	
IC	9.45	N/A	N/A
CSO	N/A	228.3	N/A
Mixture (IC : CSO = 1 : 20)	6.56	150.80	1.35
Mixture (IC : CSO = 1 : 30)	5.59	178.90	1.22
Mixture (IC : CSO = 1 : 40)	6.73	250.70	1.78
Mixture (IC : CSO = 1 : 50)	3.74	187.00	1.78
IC-MEs (IC : CSO = 1 : 20)	2.86	65.79	0.59
IC-MEs (IC : CSO = 1 : 30)	1.41	45.13	0.31
IC-MEs (IC : CSO = 1 : 40)	1.68	67.31	0.45
IC-MEs (IC : CSO = 1 : 50)	1.48	74.34	0.42

#### 3.3.3. Autophagy-mediated apoptosis effect study

Apoptosis and autophagy play important roles in the development of HCC (Gual et al., 2017). It was reported that autophagy (type II programmed cell death) and apoptosis (type I programmed cell death) respectively determine the fate of organelles and cells. There are complex interactive regulations between autophagy and apoptosis (Mukhopadhyay et al., 2014). Autophagy has dual roles in regulating cell survival and cell death. On the one hand, tumor cells could exploit autophagy to avoid chemotherapeutic drug induced-apoptosis for surviving (Katheder et al., 2017). On the other hand, autophagy also could produce tumor-suppressive effect by triggering autophagic cells death and cancer cells apoptosis (Z. Li et al., 2016; Zhang et al., 2016; Tao et al., 2017).

**Figure 6.**
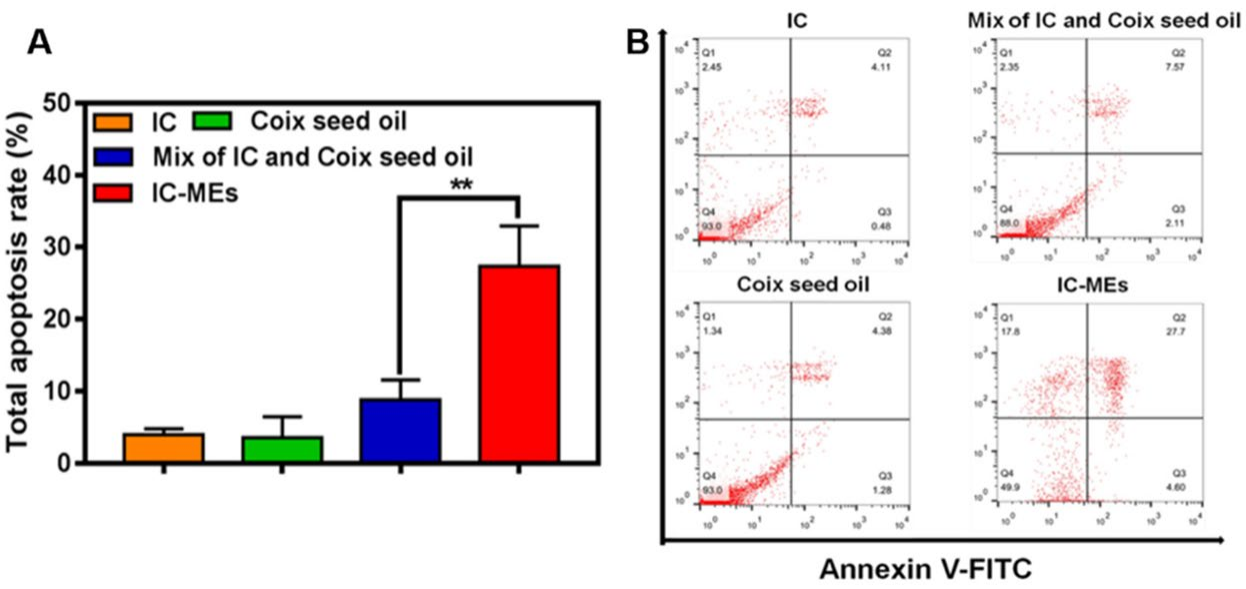
(A) Quantitative analysis and (B) apoptosis ratio of HepG2 cells treated with various formulations for 4 h. Data are represented as mean ± SD, *n* = 3, **p < .01.

The mechanism of IC-MEs-induced apoptosis of HepG2 cells was investigated in this part. Annexin V-FITC/PI detection kit was employed to investigate apoptosis in HepG2 cells caused by IC, CSO, the mixture of IC and CSO, and IC-MEs. As demonstrated in Figure 6, the apoptosis rate of IC-MEs group was 27.7%, which was 6.74- and 6.32-folds to the IC and CSO group, respectively. In addition, the apoptosis ratio of IC-MEs group was significantly higher than that in the mixture of IC and CSO group (*p* < 0.01), which may be related to the promoting internalization of microemulsion. These results confirmed that IC-MEs could significantly induce apoptosis of HepG2 cells compared with IC and CSO.

**Figure 7.**
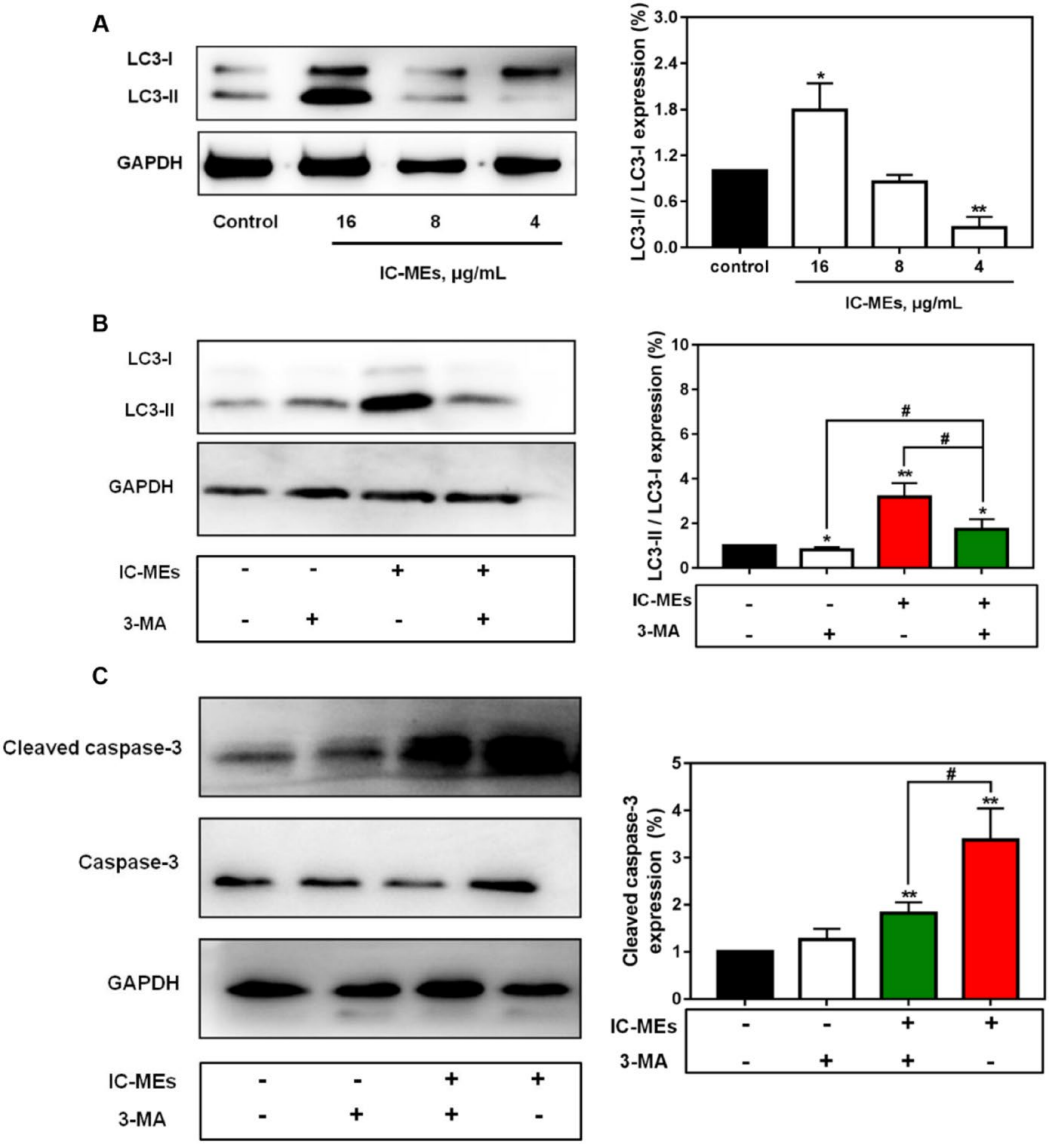
IC-MEs induce autophagy and apoptosis in HepG2 cells. (A) The expression ratio of LC3-II/LC3-I after IC-MEs treatment. (B) The expression ratio of LC3-II/LC3-I after IC-MEs co-incubated with 3-MA. (C) The expression of cleaved caspase-3 treated with different drugs administration. Data are represented as mean ± SD, *n* = 3, *p < .05, **p < .01 vs. control, ^#^*p* < .05.

Our study demonstrated that IC-MEs could induce activation of autophagy and apoptosis. We further investigated the expression of apoptosis-related proteins mediated by autophagy (Figure 7(C)). It was found that the expression of cleaved caspase-3 was dramatically upregulated by IC-MEs, suggesting IC-MEs could induce apoptosis in HepG2 cells. The quantitative analysis of apoptosis rate measured by flow cytometry also obtained similar results.

To investigate whether autophagy was involved in the antitumor mechanism of IC-MEs, we first examined the expression of autophagy-related protein LC3 II/I in response to IC-MEs (16, 8, 4 μg/mL) by western blotting. The cytoplasmic LC3 (LC3-I) will decompose a small polypeptide enzymatically and transform into an autophagosome membrane type (LC3-II) when autophagy is activated. The increase of LC3-II/LC3-I ratio reflects the enhancement of autophagic level. As shown in Figure 7(A), IC-MEs (16 μg/mL) increased the protein levels of LC3 II/I. We speculated that this increase may be related to the activation of autophagy, the blocking of autophagy degradation, and the combination of both. Therefore, the autophagy inhibitor 3-MA was used to further confirm IC-MEs-induced autophagy in HepG2 cells. As shown in Figure 7(B), the induction of autophagy by IC-MEs was partially reverted with 3-MA, demonstrating that 3-MA attenuated the formation of autophagosome and autophagy induced by IC-MEs. In addition, the combination of IC-MEs and 3-MA resulted in enhancement of LC3-II/LC3-I protein levels compared with the control group. Collectively, the result confirmed that IC-MEs induced autophagy in HepG2 cells.

To identify how autophagy and apoptosis interact to explain the synergistic antitumor effect of IC-MEs, we treated HepG2 cells with IC-MEs alone or IC-MEs together with the autophagy inhibitor 3-MA. Importantly, the combined treatment with IC-MEs and 3-MA significantly decreased cleaved caspase-3 expression, suggesting that the apoptosis of HepG2 cells was inhibited in the case of autophagy inhibition. Taken together, these outcomes revealed that IC-MEs can induce apoptosis by activating the autophagy signaling pathway in HepG2 cells.

### 3.4. Antitumor efficacy *in vivo*

**Figure 8.**
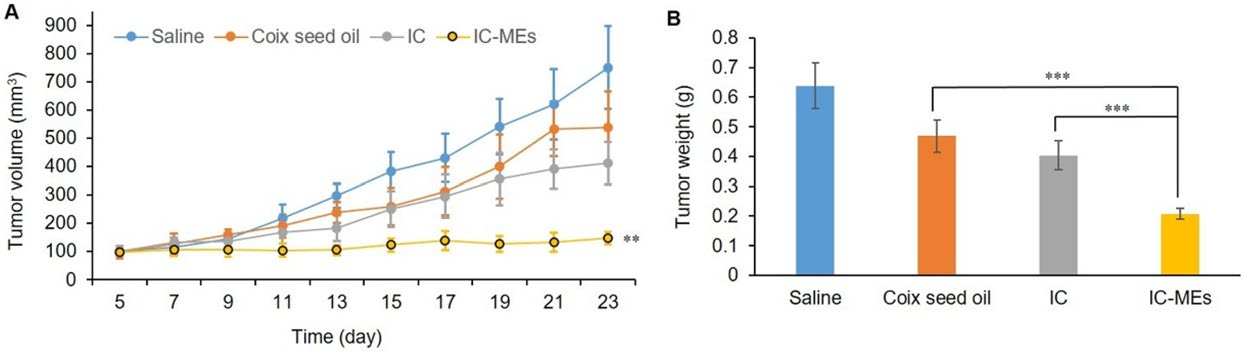
Antitumor efficacy *in vivo*. (A) Changes in tumor volume after xenograft implantation of each formulation group. Data are represented as mean ± SD, *n* = 5, compare with IC and CSO groups, IC-MEs, **p < .01. (B) Tumor weight of each formulation group at the end of the treatment. Data are represented as mean ± SD, *n* = 5, ***p < .001.

In this part, the antitumor efficacy of IC-MEs *in vivo* was evaluated in BALB/c nude mice. As can be seen from the curve of tumor volume and tumor weight (Supplementary Figure S1, Figure 8), tumor growth was inhibited in each administration group. Compared with the IC group and CSO group, IC-MEs showed a significant potent inhibition of tumor growth. The tumor volume and weight of the mice treated with IC-MEs were obviously lower than other groups. The tumor inhibition ratio of the IC-MEs group was 69.82%, which was 2.14- and 1.78-fold those of the CSO group and IC group, respectively. It could be inferred that the contribution of IC-MEs to the antitumor activity may be from the synergistic effect of IC and CSO and the sustained release of microemulsion.

### 3.5. *In vivo* pharmacokinetic study

**Figure 9.**
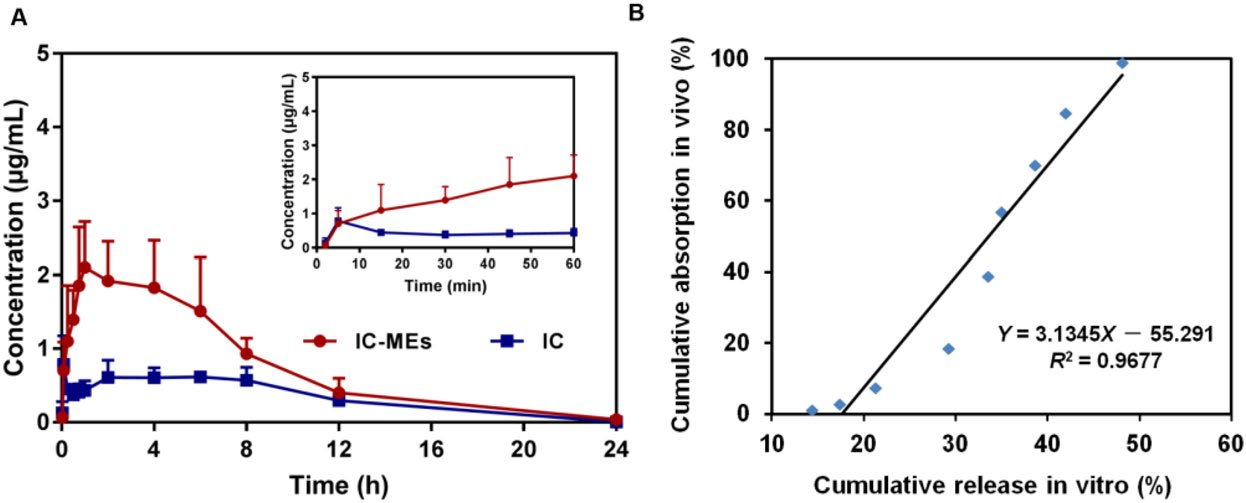
(A) Concentration–time curve of IC-MEs and IC in rat plasma after intraperitoneal administration. Data are represented as mean ± SD, *n* = 6. (B) IVIVC of IC-MEs.

The plasma pharmacokinetic of IC-MEs was conducted on male SD rats by HPLC–QDa method. The precision, accuracy, extraction recovery, and stability of IC in rat plasma and liver are displayed in Table 6. The results showed that it met the requirement of biological sample determination. Pharmacokinetic analysis was carried out by the non-atrioventricular model of DAS 2.0 software, so as to obtain main the pharmacokinetic parameters. The mean plasma concentration–time curves for IC-MEs and IC are shown in Figure 9(A) and the pharmacokinetic values were represented in Table 7. Compared with IC group, IC-MEs showed a significant increase in the mean value of *C*_max_, AUC_(0-_*_t_*_)_, AUC_(0-∞)_, and *t*_1/2_, as well as a significant decrease of *T*_max_ and CL (*p* < .001). IC is a BCS II class drug, so the dissolution rate is the rate-limiting step for its absorption. From these pharmacokinetic results, it is obvious that microemulsion could improve the absorption rate and bioavailability of drugs *in vivo*, and reduce the elimination rate of drugs in plasma and prolong the retention time in rat blood.

**Table 6.** Precisions, accuracy, extraction recovery and stability for the determination of icaritin in different samples.

Verification indicators		Rat plasma (*n* = 6, ng/mL)			Rat liver (*n* = 6, ng/g)		
		49	196	1256	39	625	15,200
Intra-day precision	RSD (%)	5.48	12.80	6.40	9.40	10.01	7.15
Inter-day precision	RSD (%)	8.93	7.25	3.61	7.09	8.20	6.14
Accurcy	RE (%)	0.99	2.20	7.28	–1.30	–1.22	–6.58
Extraction recovery	Mean	76.92	87.92	81.52	83.08	86.74	82.94
	RSD (%)	7.66	11.88	8.31	11.79	4.32	4.87
Stability, 25 °C, 8 h^a^	RE (%)	3.09	–3.11	7.00	8.61	9.13	10.27
Stability, 4 °C, 24 h^b^	RE (%)	9.13	5.09	1.20	6.09	13.87	9.09
Stability, 3 times, –80 °C/25 °C^c^	RE (%)	7.54	–4.63	–3.10	11.52	10.25	9.15
Stability, –20 °C, 2 weeks^d^	RE (%)	9.08	–1.76	–6.74	12.99	7.87	9.00

RE: relative error; RSD: relative standard deviation.

^a^ The quality control samples were placed at 25 °C for 8 h.

^b^ The quality control samples were placed at 4 °C for 24 h.

^c^ The quality control samples were repeated three times at –80 °C/25 °C.

^d^ The quality control samples were placed at –20 °C for 2 weeks.

**Table 7.** Pharmacokinetic parameters of IC-MEs and IC.

Pharmacokinetic parameters	IC (*n* = 6)	IC-MEs (*n* = 6)
*C*_max_ (mg/L)	0.92 ± 0.29	2.31 ± 0.69**
*T*_max_ (h)	3.36 ± 3.23	2.50 ± 2.07
AUC_0–_*_t_* (mg/L h)	8.12 ± 0.58	18.18 ± 4.73***
AUC_0–∞_ (mg/L h)	8.15 ± 0.57	18.38 ± 4.72***
*t*_1/2_ (h)	2.53 ± 0.24	3.51 ± 0.29***
CLz/*F* (L/h/kg)	7.40 ± 0.50	3.50 ± 1.11***
*F*_rel_ (%)	–	225.58

^**^p < .01, ^***^p < .001 vs. IC.

Data are represented as mean ± SD, *n* = 6.

Furthermore, the *in vivo* and *in vitro* correlation between the cumulative release rate *in vitro* (%) and the absorption fraction *in vivo* (%) was established through linear fitting, and the results are shown in Figure 9(B). *R*^2^ is a standard for evaluating the degree of fitting of the linear regression equation, and *R*^2^ > 0.95 indicates a good correlation *in vivo* and *in vitro*. As shown in Figure 9(B), the *in vitro* release behavior of IC-MEs had a good correlation with the blood concentration *in vivo*.

### 3.6. Tissue distribution study

**Figure 10.**
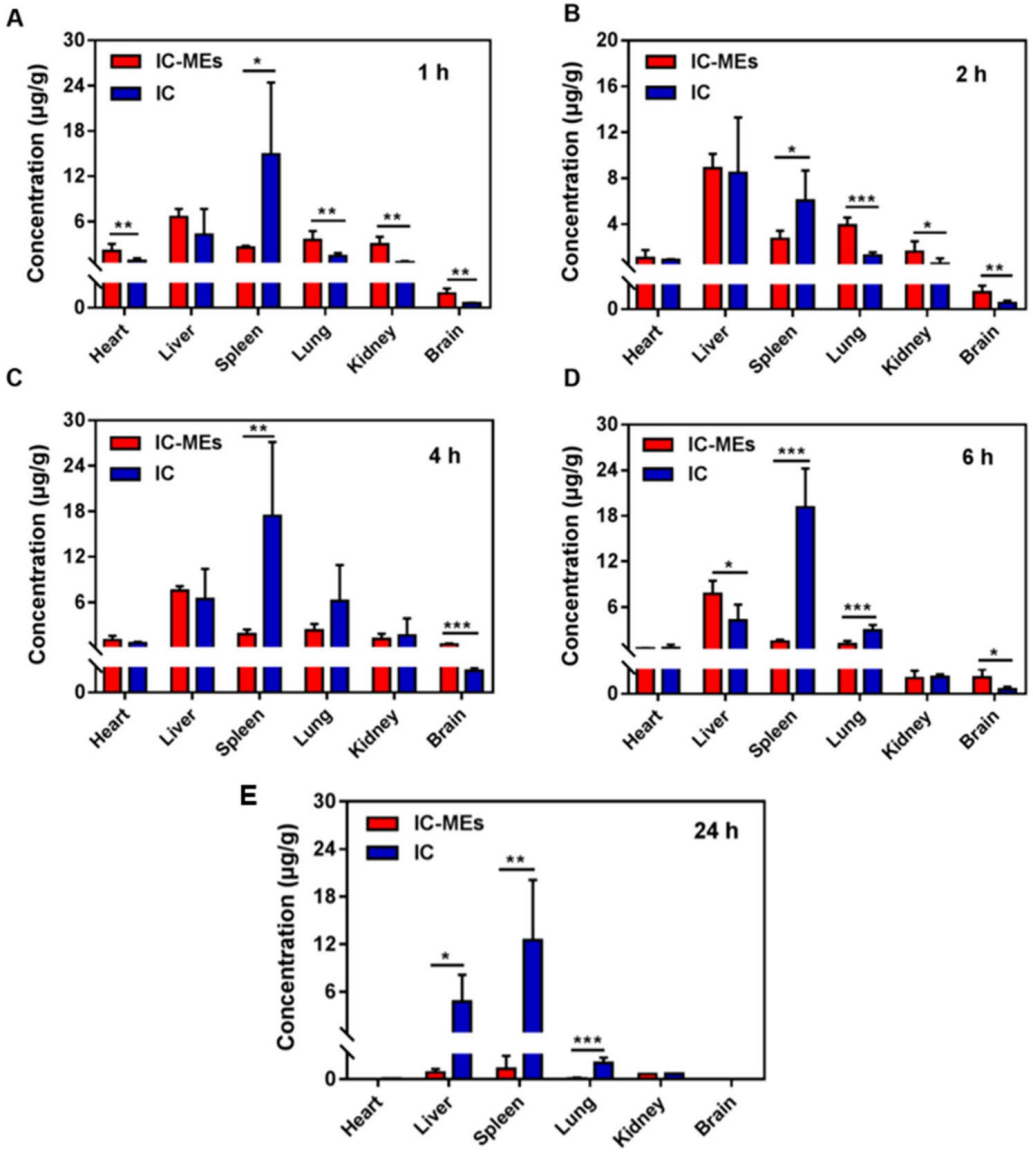
Distribution of IC-MEs and IC in various tissues of rats. (A) After 1 h of administration. (B) After 2 h of administration. (C) After 4 h of administration. (D) After 6 h of administration. (E) After 24 h of administration. Data are represented as mean ± SD, *n* = 5, *p < .05, **p < .01, ***p < .001.

The distribution of IC-MEs in liver, heart, spleen, lung, kidney, and brain were studied. The concentration–time profiles of IC-MEs and IC in rat tissues are shown in Figure 10. AUC_0-6_ values in various tissues of IC-MEs and IC are calculated in Table 8. It could be seen that both IC-MEs and IC were differently distributed in various tissues. The AUC_0-6_ values (µg/g h) of the main tissues in the IC-MEs group were in the order of liver, lung, spleen, kidney, heart, and brain, with liver accounting for the highest proportion, indicating that IC-MEs had strong liver targeting, while the AUC_0-6_ values (µg/g h) in the major tissues of rats in the IC group were, from high to low, spleen, liver, lung, kidney, heart, and brain, and most of the drug was concentrated in the spleen. The results indicated that the microemulsion could change the tissue distribution ratio of IC *in vivo*, significantly improve the drug distribution in liver, thus. As shown in Figure 10, compared with the IC group, IC-MEs group were rapidly eliminated in heart, spleen, lung, kidney, and brain after 6 h administration, which indicated that IC-MEs could significantly reduce the retention and accumulation of IC in tissues. To sum up, IC-MEs could obviously benefit the treatment of liver cancer and reduce the potential toxic and side effects of drugs on normal tissues.

**Table 8.** AUC_0–6_ values in various tissues of IC-MEs and IC.

Tissues	AUC_0–6_ (µg/g h)	
	IC-MEs (*n* = 5)	IC (*n* = 5)
Liver	43.32	34.13
Heart	6.27	3.92
Spleen	11.68	77.87
Lung	15.09	18.57
Kidney	7.99	4.96
Brain	1.97	0.50

### 3.7. Safety evaluation

**Figure 11.**
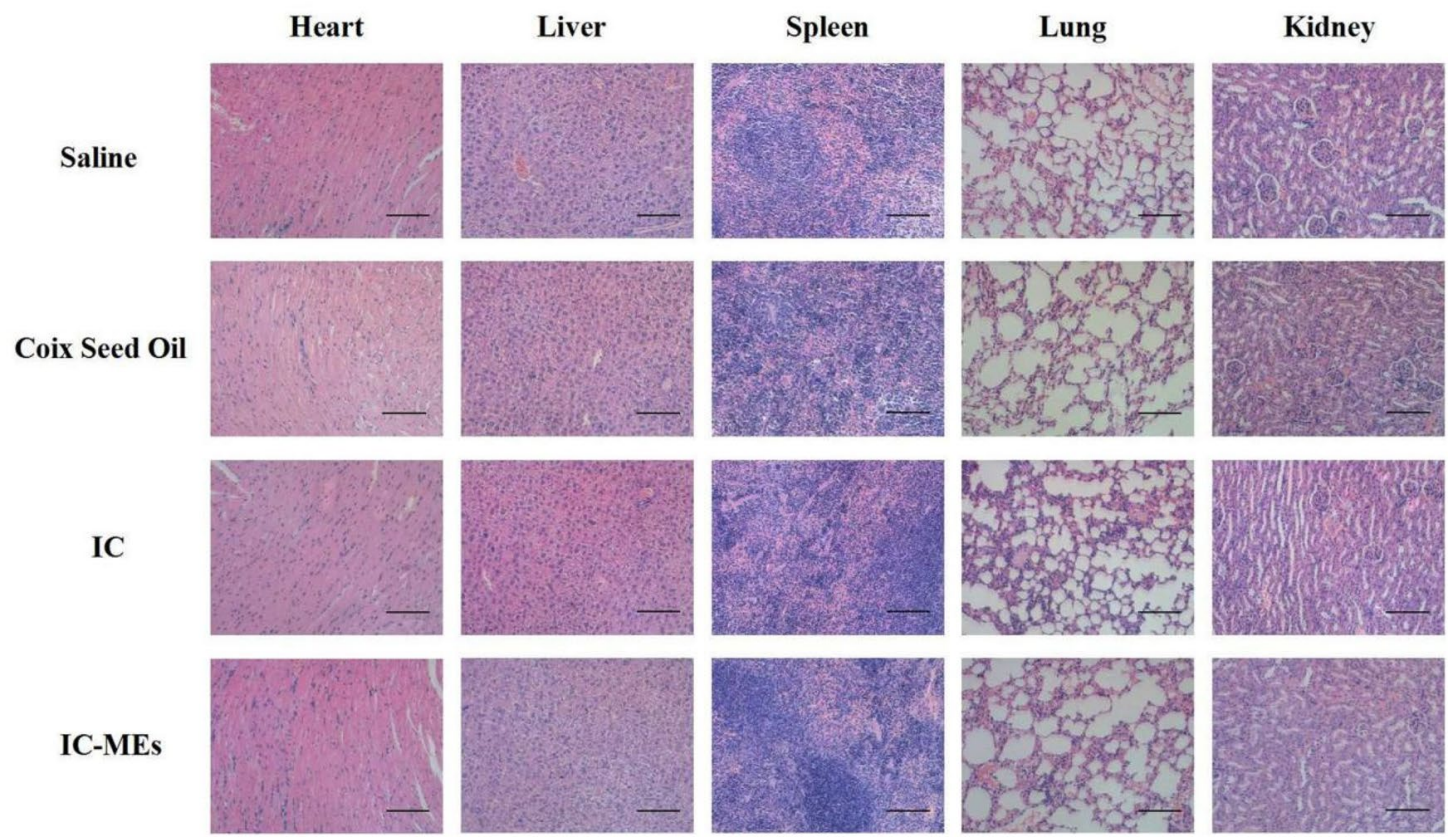
H&E staining of heart, liver, spleen, lung, and kidney of various formulation groups. Scale bar: 100 μm.

To evaluate the systemic toxicity against mice treated with IC-MEs, some important indicators for safety evaluation including body weight of nude mice and histopathology of main organs has been evaluated during and after antitumor treatment. As seen from Supplementary Figure S2, there was no significant change of body weight during the treatment, suggesting IC-MEs has a relatively low systemic toxicity. According to the results of H&E staining of major organs in each group (Figure 11), no obvious cell necrosis or inflammation in each group were observed, indicating that no major organ of mice treated with IC-MEs was damaged. All the data suggest that IC-MEs have a nice biosafety and could be used as a safe nanomedicine *in vivo* for the treatment of liver cancer.

## 4. Conclusion

In this study, we developed a dual-component microemulsion composed of IC and CSO (IC-MEs) for the treatment of liver cancer. The preparation process of IC-MEs was optimized by Box–Behnken design for industrialization and the physicochemical properties were further evaluated. IC-MEs exhibited significant synergistic antitumor effect and enhanced apoptosis induction in HepG2 cells, and the further study indicated that IC-MEs could induce caspase-dependent apoptosis by activation of autophagy in HepG2 cells. Pharmacokinetic and biodistribution study indicated that IC-MEs could effectively improve the bioavailability of IC and reduce the distribution of IC in non-liver organs, and the safety evaluation showed that IC-MEs was safe enough for clinical application. This study offers a synergistic antitumor technology and strategy of IC and CSO to effectively improve the anti-liver efficacy of active ingredients from traditional medicines.

## Ethics approval

All experimental protocols and animal handling procedures were approved by the Animal Ethic Committee of Affiliated Hospital of Integrated Traditional Chinese and Western Medicine, Nanjing University of Chinese Medicine (Approval No.: AEWC20181207-68) and were performed in accordance with institutional, national and international guidelines for animal care and welfare. All the animals were raised and bred under specific pathogen-free conditions in the Animal Center of Affiliated Hospital of Integrated Traditional Chinese and Western Medicine, Nanjing University of Chinese Medicine. All mice were kept on a standard 12 h light-to-dark cycle with ad libitum access to food and water, and were anesthetized with 1.5% isoflurane using a Rodent Anesthesia Machine. All the research meets ethical guidelines and adheres to the legal requirements of the study country.

## Supplementary Material

Supplemental Material
