# Supplementary material for: An icaritin-loaded microemulsion based on coix oil for improved pharmacokinetics and enhanced antitumor efficacy
Source: Drug Deliv. 2022 Nov 29;29(1):3454–66. doi: 10.1080/10717544.2022.2147601 (PMC9848417; doi:10.1080/10717544.2022.2147601)
Supplement: Supplemental Material [file IDRD_A_2147601_SM4545.docx]

**
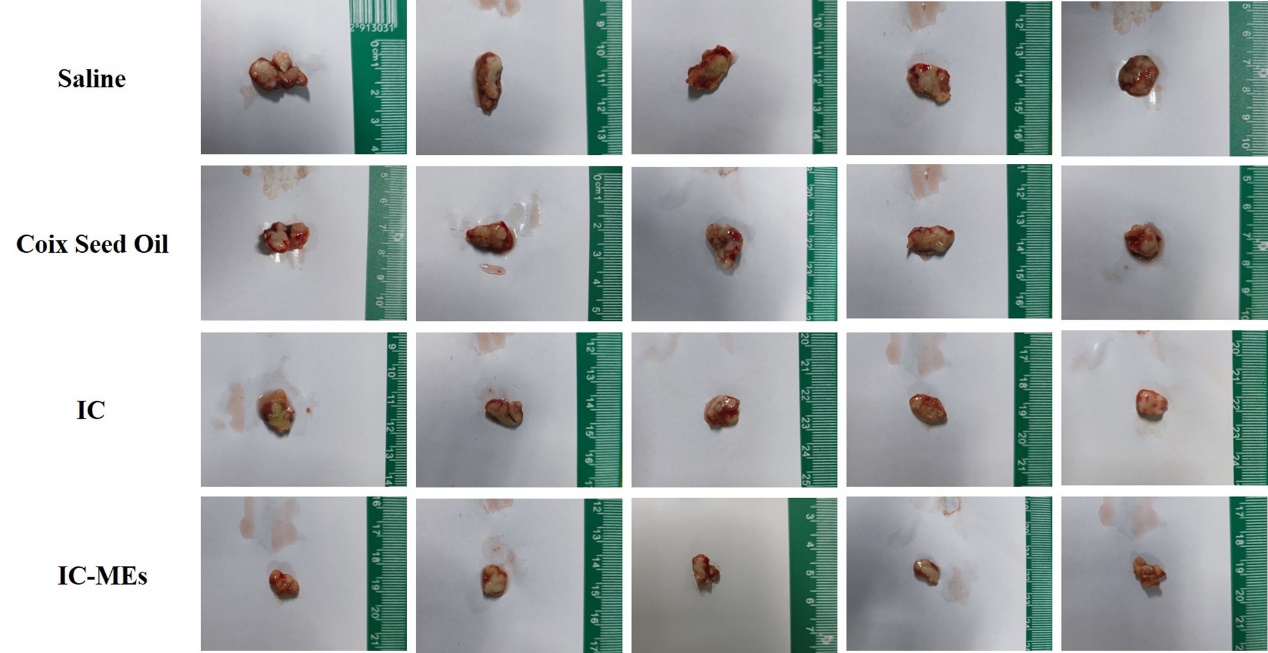
**

Figure S1 The harvested tumors of various groups after antitumor treatment (n=5)


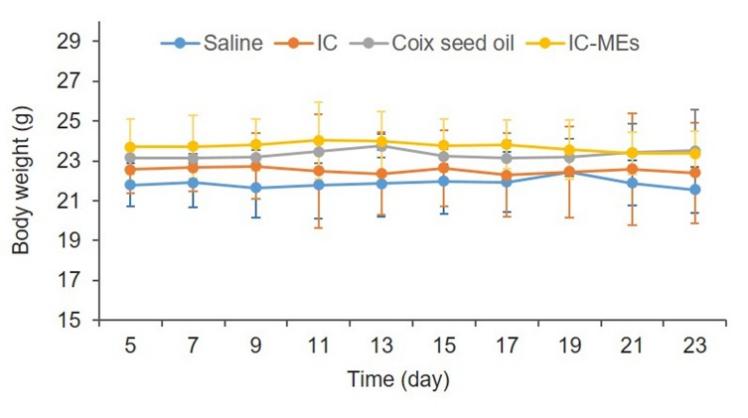


Figure S2 Weight change of nude mice after xenograft. Data are represented as mean ± SD, n = 5


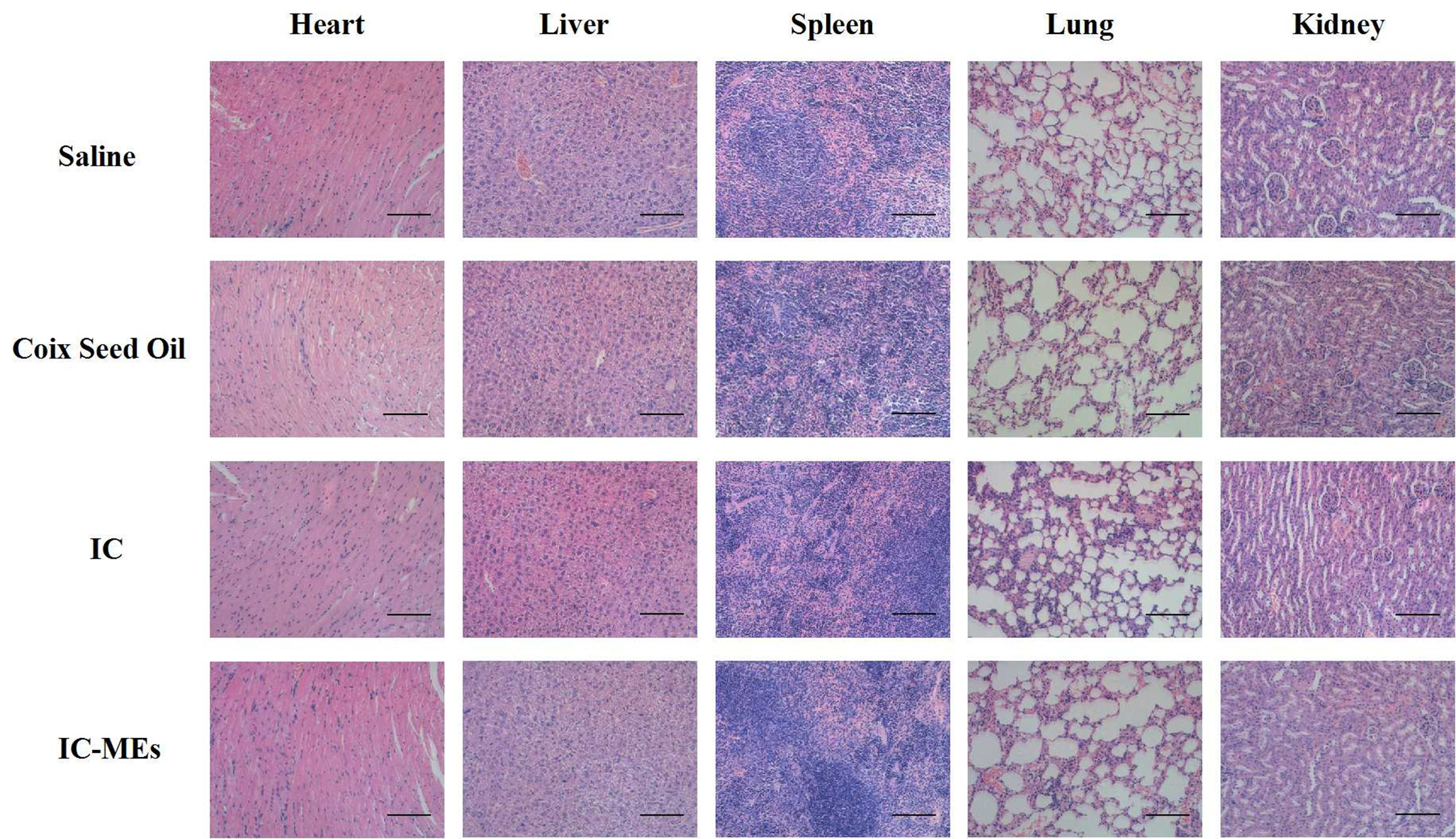


Figure 11 H&E staining of heart, liver, spleen, lung and kidney of various formulation groups. Scale bar: 100 μm
